# Nano-Engineered Cetuximab-Copper Complexes for Targeted Drug Delivery in Head and Neck Cancer

**DOI:** 10.2174/0115672018373037250821092024

**Published:** 2025-08-28

**Authors:** Majed S. AlFayi

**Affiliations:** 1 Department of Clinical Laboratory Sciences, College of Applied Medical Sciences, King Khalid University, Abha, Saudi Arabia

**Keywords:** Cetuximab, drug delivery, nanoparticles, head and neck cancer, metal, copper

## Abstract

**Introduction:**

Head and neck squamous cell carcinomas (HNSCCs) require precise treatments. Cetuximab (Ceb) targets EGFR, and copper (Cu) compounds show promise in cancer therapy. This study investigates Ceb-Cu-p-NC, a nanoengineered drug delivery system, designed for enhanced HNSCC treatment. The objective of this study is to evaluate the potential of Ceb-Cu-p-NC in HNSCC treatment.

**Methods:**

Cu precursor, Ceb, poloxamer-407, and hyaluronic acid were used to synthesize Ceb-Cu-p-NC. Fluorescence microscopy and UV spectrophotometry were utilized to determine Ceb integration efficiency, cellular interactions, and drug concentration. Drug release was assessed *via in-vitro* studies at pH 5.4 and 7.4. Studies using A-253 cell lines were conducted to analyze cytotoxicity, viability, apoptosis, and cell cycle arrest.

**Results:**

In this study, Ceb-Cu-p-NC showed size reduction (85-120 nm) and zeta potential shift. The Ceb integration was 34.92% with 82.5% entrapment efficiency. Cytotoxicity studies revealed enhanced efficacy (IC50: 27.55 mg/mL - 51.47 mg/mL). Flow cytometry showed significant apoptosis and S-phase cell cycle arrest, with statistically significant results (*p* < 0.05).

**Discussion:**

Ceb conjugation to Cu-p-NC enhanced nanoparticle stability, reduced surface charge, and enabled targeted, controlled drug release. The formulation showed superior cytotoxicity, apoptosis induction, and S-phase arrest in A-253 cells compared to free Ceb, highlighting its potential as an effective targeted therapy for head and neck cancer.

**Conclusion:**

Ceb-Cu-p-NC demonstrates targeted efficacy against HNSCCs, with controlled release, increased cytotoxicity, and apoptosis.

## 1 INTRODUCTION

Cancer remains a major global health concern, with an estimated 2,001,140 new cases and 611,720 deaths projected in the U.S. in 2024. While cancer mortality has declined by 33% since 1991, certain cancers, such as breast and colorectal cancer, continue to rise, especially among younger individuals [1, 2]. Breast cancer alone accounts for 310,720 new cases and 42,250 deaths this year. Additionally, cancer incidence in women has been increasing due to the growing prevalence of breast, uterine, and melanoma cases. These trends emphasize the urgent need for ongoing research, improved treatments, and early detection strategies to reduce cancer burden and improve patient survival [3, 4].

Cancer treatment has advanced over the years, moving beyond traditional methods like surgery, chemotherapy, and radiation therapy [5]. At the same time, surgery is effective for removing localized tumors, chemotherapy and radiation target rapidly growing cancer cells. Modern approaches focus on personalized medicine, where treatments are tailored based on a patient’s genetic profile. Targeted therapies reduce harm to healthy cells, and immunotherapy strengthens the body’s natural defense against cancer [6]. New developments, such as mRNA vaccines, nanotechnology for precise drug delivery, and CAR-T cell therapy, offer promising results. However, challenges remain, underscoring the need for ongoing research to enhance cancer treatment outcomes [7].

Head and neck squamous cell carcinomas (HNSCCs) present a diverse array within epithelial malignancies affecting anatomical regions such as the nasopharynx, oral cavity, salivary glands, oropharynx, and larynx. The primary etiological factors include prolonged alcohol consumption and tobacco use, with a unique case intricately linked to human papillomavirus (HPV) infection. Globally, HNSCCs have significant prevalence, with the year 2022-23 documenting over 0.93 million new cases and approximately 0.54 million fatalities [8, 9]. This malignancy accounts for approximately 6.78% of all cancer cases and over 3.5% of total cancer-related deaths [9], emphasizing its multifaceted nature and the intricate interplay of diverse risk factors and underlying biological mechanisms in its pathogenesis [10, 11].

Crafting interventions tailored for HNSCCs involves navigating various considerations, such as disease stages, functional aspects, anatomical subsites, and patient desires. The distinct natural histories of HPV-related and tobacco-related HNSCC necessitate a nuanced approach, particularly acknowledging the challenges inherent in delivering intensive therapy to elderly patients [12].

Standard treatment protocols for HNSCCs seamlessly integrate surgical intervention, radiotherapy, and chemotherapy, chosen meticulously based on comprehensive assessments covering the patient's condition, anatomical specifics, disease extent, clinical nuances, functional considerations (including the preservation of speech and swallowing functions), and patient preferences [13, 14]. This personalized and multidisciplinary paradigm underscores the inherent complexity in managing HNSCC, emphasizing the need to tailor treatments to the distinctive characteristics and circumstances of each patient [15].

Cetuximab (Ceb), a chimeric human-murine IgG1 monoclonal antibody, holds a pivotal role in this landscape by inhibiting Epidermal Growth Factor Receptor (EGFR) activation [16, 17]. Its multifaceted impact involves disrupting cell cycle progression, inhibiting angiogenesis, and demonstrating efficacy in conjunction [18] with other chemotherapeutic drugs for recurrent/metastatic conditions, as validated by FDA approval [19-21].

The field of metal-based compounds in cancer therapeutics faces the challenge of establishing a clear therapeutic-toxicity threshold [22]. Addressing this, contemporary research endeavors into synthesizing metal compounds strive to redesign existing chemical structures or formulate entirely new compounds [23], with an emphasis on improved safety profiles and heightened cytotoxic characteristics [24]. This innovative approach seeks to optimize the therapeutic efficacy of metal-based compounds while mitigating potential toxic effects, responding to the critical need for a nuanced and targeted application of these compounds in cancer treatment [25, 26].

Copper (Cu), recognized for its vital role in various physiological processes, assumes a dual role of benefit and detriment to cells due to its distinctive redox properties [27]. Excessive copper levels exceeding cellular requirements lead to cytotoxicity and cell death, while copper deficiency disrupts absorption and transport, causing abnormal distribution within cells [28, 29]. The heightened demand for copper by proliferating tumors, coupled with diagnostic and treatment methodologies associated with copper, holds relevance for tumor suppression [30, 31].

Investigations into how copper and its complexes induce autophagy and apoptosis in tumor cells mark a significant stride in advancing our understanding of the intricate interplay between copper and tumor cells. This knowledge contributes to innovative approaches in cancer diagnosis and treatment [32].

Nanoparticles play a vital role in the field of cancer therapy, especially in advancing intelligent drug delivery systems, forming the basis for this investigation [33]. Novel approaches center on the integration of diagnostic and therapeutic components into a unified nanosystem, establishing a platform for cancer treatment guided by imaging, addressing the shortcomings of traditional clinical methods [34, 35]. Various theranostic nanosystems, spanning liposomes, polymeric nanoparticles, graphene oxide, carbon nanotubes, nano-micelles, dendrimers, and metal-based nanomaterials, hold potential in improving the pharmacokinetics, pharmacology, biomedical performance, and bioavailability of chemotherapeutic agents [36, 37]. Despite the proven therapeutic effectiveness and real-time imaging capabilities, the efficacy of these theranostic NPs against tumors faces obstacles such as inadequate drug dosages and weak imaging signals, stemming from the suboptimal accumulation of NPs at the tumor site during *in vivo* circulation [38]. Thus, the essential focus lies in optimizing the functions of theranostic nano-systems to enhance their internalization within tumors while avoiding nonspecific interactions with normal tissues. This strategic optimization can significantly boost the overall efficiency of the theranostic approaches in cancer treatment [39, 40].

The current research focuses on assessing the impact of integrating Ceb with Cu complexes in nanoengineered targeted drug delivery on head and neck cancer therapeutics. This investigation aims to reveal how the fusion of Ceb, a monoclonal antibody, with Cu complexes has transformed the field of nanoengineering. The study specifically aims to uncover the increased efficacy and precision achieved through this innovative approach in delivering therapeutic agents to affected regions in head and neck malignancies. By capitalizing on the unique properties of Ceb-Cu complexes, the research strives to contribute to the transformation and optimization of targeted drug delivery systems, paving the way for more effective and personalized treatment strategies in HNSCCs.

## 2 MATERIALS AND METHODS

### 2.1 Materials

Copper chloride (97%) sourced from Merck Ltd. serves as the precursor for nanocrystal formulation. Cetuximab, acquired from Selleck USA, serves as both a surface modifier and imparts a chemotherapeutic effect. Poloxamer 407, obtained from Specialized Solutions, USA, functions as a stabilizer, while hyaluronic acid from Sigma-Aldrich is used for cross-linking purposes. All other chemicals were obtained commercially and met analytical-grade standards.

### 2.2 Synthesis of Cu Modified Polymeric Nanocrystal (Cu-p-NC)

The Cu-p-NC was formulated by the precipitation polymerization method. Anhydrous copper chloride (0.5mg/mL, 2.5 mL) and hyaluronic acid (1.25%) (1:4) were cross-linked under reduced pressure at an optimum temperature of 60°C. The mixture was stirred for 45 minutes in the presence of propylene glycol. After cooling to room temperature, the intermediate product was isolated through centrifugation, washing, and vacuum drying [41]. Subsequently, a 2.8% magnesium hydroxide solution (0.9 mol∙mL−1.5) was added and heated at 85°C for 30 hours. Before this, aqueous solutions of poloxamer 407 (0.053 g/mL, 12 mL) and deionized water (60 mL) were mixed under continuous stirring for 12 hours. The final product was collected by ultra-centrifugation, washed with deionized water, freeze-dried, and stored at 4°C [42].

### 2.3 Loading of Ceb into Cu-p-NC

Cu-p-NC was loaded with Ceb at a concentration of 5mg/mL (20 μL), using an equivalent mass, and sink with phosphate-buffered solution (PBS) (pH 7.0), comprising sodium phosphate (Na_3_PO_4_, 10mM) and sodium chloride (NaCl, 145mM) (pH 7.2). Following 24 hours of swirling in darkness, the product was collected through ultra-centrifugation, leaving the unloaded Ceb in the supernatant. The mass of Ceb loaded into Cu-p-NC was determined by subtracting the supernatant Ceb from the total Ceb measured using a fluorescence spectrophotometer. Fig. (**1**) shows the synthesis and loading of Ceb into Cu-p-NC.

### 2.4 Analysis of Ceb-Cu-p-NC Characteristics

Evaluation of Polydispersity Index (PDI), particle size, and Zeta Potential involved dynamic light scattering (DLS), conducted with an automated inspection system, Malvern Zeta-Sizer. Morphological examination utilized a transmission electron microscope (EM900, Germany) to visualize the structure of Ceb-Cu-p-NC [43].

### 2.5 *In-vitro* Drug Release and Entrapment Efficacy of Ceb in Ceb-Cu-p-NC

An in-vitro study on the release of Ceb from Ceb-Cu-p-NC was conducted where a dialysis procedure was performed. Each 2 mL sample was enclosed in a dialysis membrane (a 12 kDa) and immersed in 50 mL of PBS-SDS solution 0.5%w/v (37°C), maintaining pH levels of 5.4 and 7.4 for 72 hours [44]. At designated intervals, 1 mL of the sample was withdrawn, and assessed over a UV light spectrophotometer and the released pattern was recorded against time at 590 nm. A parallel control experiment was executed using a free drug.

The entrapment efficiency of Ceb-Cu-p-NC formulations was evaluated using ultra-filtration at 4000×g for 20 minutes. This process separated the entrapped Ceb from the non-entrapped Ceb over Cu-p-NC. The amount of Ceb was measured using a UV-Visible spectrophotometer at 590 nm with a specific equation (1) [45]:

EE% = [(Ceb _(Ceb-Cu-p-NC)_ – Ceb _(untrapped)_) / Ceb _(Ceb-Cu-p-NC)_] X 100 (1)

### 2.6 Stability Assessment

The stability evaluation involved subjecting both Cu-p-NC and Ceb-Cu-p-NC formulations to storage conditions at 25°C and 4°C, maintaining a relative humidity of 60 ± 5% for a duration of three months. After the storage period, the samples underwent analysis for particle size, PDI, and the concentration drug up to three months to gauge the endurance of the formulations [46].

### 2.7 Cell Line Cultivation

In cultivating the A-253 cell line, cells from the sub-maxillary salivary gland of the head and neck region were obtained from the ATCC Cell Bank. The cells were cultured under controlled conditions that are as follows: optimum temp, 5% CO_2_, in RPMI-1640 medium with a 10:1 ratio of FBS and streptomycin. The cells underwent specific steps: medium withdrawal, 0.25% trypsin-EDTA detachment, re-suspension in a complete growth medium, trypan blue staining, and quantification using a hemocytometer [46].

### 2.8 Assessing Cytotoxicity and Viability

To evaluate the drug delivery system's cytotoxicity and viability, the MTT assay was conducted on the A-253 cell line. Seeding 2 × 10^5 cells per well, they were exposed to varied concentrations of Ceb, Cu-p-NC, and Ceb-Cu-p-NC for up to 72 hours. Absorbance values at 570 nm after MTT treatment indicated cell viability, with quantification ensuring a thorough evaluation.

### 2.9 Assessing Apoptosis *via* Flow Cytometry

The induction of apoptosis in A-253 cells by the drug delivery system was assessed through flow cytometry. Following a 48-hour exposure to IC50 concentrations, cells underwent harvesting, dual PBS washes, and suspension adjustment to 5 × 10^5 cells/mL before seeding onto 6-well plates in 1X binding buffer. Employing the Apoptosis Quantitation Kit, the cells underwent staining with PI (propidium iodide) and V-FITC-annexin. An FACS flow cytometer was used to comprehensively analyze the percentages of apoptotic and necrotic cells.

### 2.10 Analyzing Cell Proliferation Through Cell Cycle Analysis

Cell proliferation was evaluated by staining DNA content with propidium iodide (PI) in 6-well plates. After drug-loaded patch treatment, cells underwent fixation, PI staining, and flow cytometry analysis. This thorough process was repeated thrice for reliability, using the IC50 concentration for consistency and accuracy.

### 2.11 Statistical Assessment: *In vitro* Ceb Release and Cell Cytotoxicity

*In vitro* Ceb release was thoroughly analyzed using one-way ANOVA. Simultaneously, MTT assay results underwent detailed evaluation, including post hoc analysis with Dunnett’s test. All experiments were conducted in three independent sets, each performed in triplicate. Statistical significance was determined at *p* < 0.05.

## 3 RESULTS AND DISCUSSION

### 3.1 Optimization of Cu-p-NC and Ceb-Cu-p-NC

The successful conjugation of Ceb onto Cu-p-NC was accomplished through meticulously executed standard protocols, ensuring the precision and reproducibility of the synthesis. This conjugation aimed to harness the unique properties of Ceb, specifically its tumor-targeting capabilities, to enhance the therapeutic potential of the resulting Ceb-Cu-p-NC.

A strategic choice was made to employ FITC-conjugated rabbit anti-human IgG as a probing agent to assess the successful conjugation [47]. The rationale was that this probe could selectively bind to the surface-conjugated Ceb, inducing the aggregation of neighboring Cu-p-NC. The ensuing aggregation phenomenon was skillfully visualized and analyzed using advanced fluorescence microscopy techniques [48]. Fig. (**2A**) illustrates the experiment’s outcomes, showing distinct green fluorescence in the Ceb-Cu-p-NC aggregates. Notably, the absence of detectable fluorescence in the Cu-p-NC control group served as a clear indicator of the specificity and success of the Ceb conjugation.

The fluorescence observations were further corroborated by advanced imaging techniques such as TEM, shown in Fig. (**2B**). Fig. (**2C**) provides a detailed glimpse into the nano-sized nature of both Cu-p-NC and Ceb-Cu-p-NC, emphasizing a homogenous diameter distribution ranging from 85 to 120 nm. This analysis also revealed a noteworthy reduction in the nanoparticles' size upon the introduction of Ceb into the Cu-p-NC matrix, suggesting a direct influence of Ceb on the nanostructure [49].

Zeta potential measurements were conducted to assess the surface charge characteristics of the prepared nanoparticles [50, 51]. The Cu-p-NC exhibited a distinct surface charge of -27 ± 5 mV, indicative of its inherent electrostatic properties. Following the conjugation with Ceb, this surface charge experienced a substantial reduction, converging to a mean value of -11 ± 5 mV in the resulting Ceb-Cu-p-NC. The observed changes in zeta potential were attributed to surface modifications induced by Ceb linkage. It is worth noting that these changes are not solely attributed to the increased molecular weight but also to the shielding effect induced by the positive charge of Ceb, hindering the inherent negative charges of Cu-p-NC.

Simultaneously, an exploration of the PDI provided insights into the uniformity of particle size distribution. The PDI values, ranging from 0.297 to 0.365 for Cu-p-NC and Ceb-Cu-p-NC, respectively, indicated that the surface modification with hydrophilic Ceb potentially contributed to an improved dispersion of Cu-p-NC in aqueous environments.

Finally, to quantitatively assess the degree of Ceb conjugation on Ceb-Cu-p-NC, a UV spectrophotometer was employed, yielding a precise determination of 34.92% as depicted in Fig. (**2D**). This quantitative analysis further affirmed the successful integration of Ceb onto the nanoparticles' surface, providing a comprehensive characterization of the synthesized Ceb-Cu-p-NC with implications for enhanced tumor-targeted drug delivery applications.

### 3.2 *In-vitro* Release Ceb from Ceb-Cu-p-NC

The Ceb-Cu-p-NC represents a groundbreaking approach to address the limitations of current drug delivery systems for HNSCCs. The primary challenge faced by existing systems lies in achieving selective drug release within tumor cells, leading to severe *in vivo* side effects [52, 53]. Ceb-Cu-p-NC innovatively circumvents this issue by employing a dual-responsive drug release profile. This sophisticated mechanism involves ligand exchange triggered by the heightened concentration of glutathione (GSH) within tumor cells, enabling the specific release of Ceb [54]. Subsequently, the departure of surface-bound Ceb facilitates the release of encapsulated Cu-p-NC. This dual-responsive strategy aims to enhance drug delivery selectivity while mitigating adverse effects during *in vivo* applications [55, 56].

**Fig. (2):**
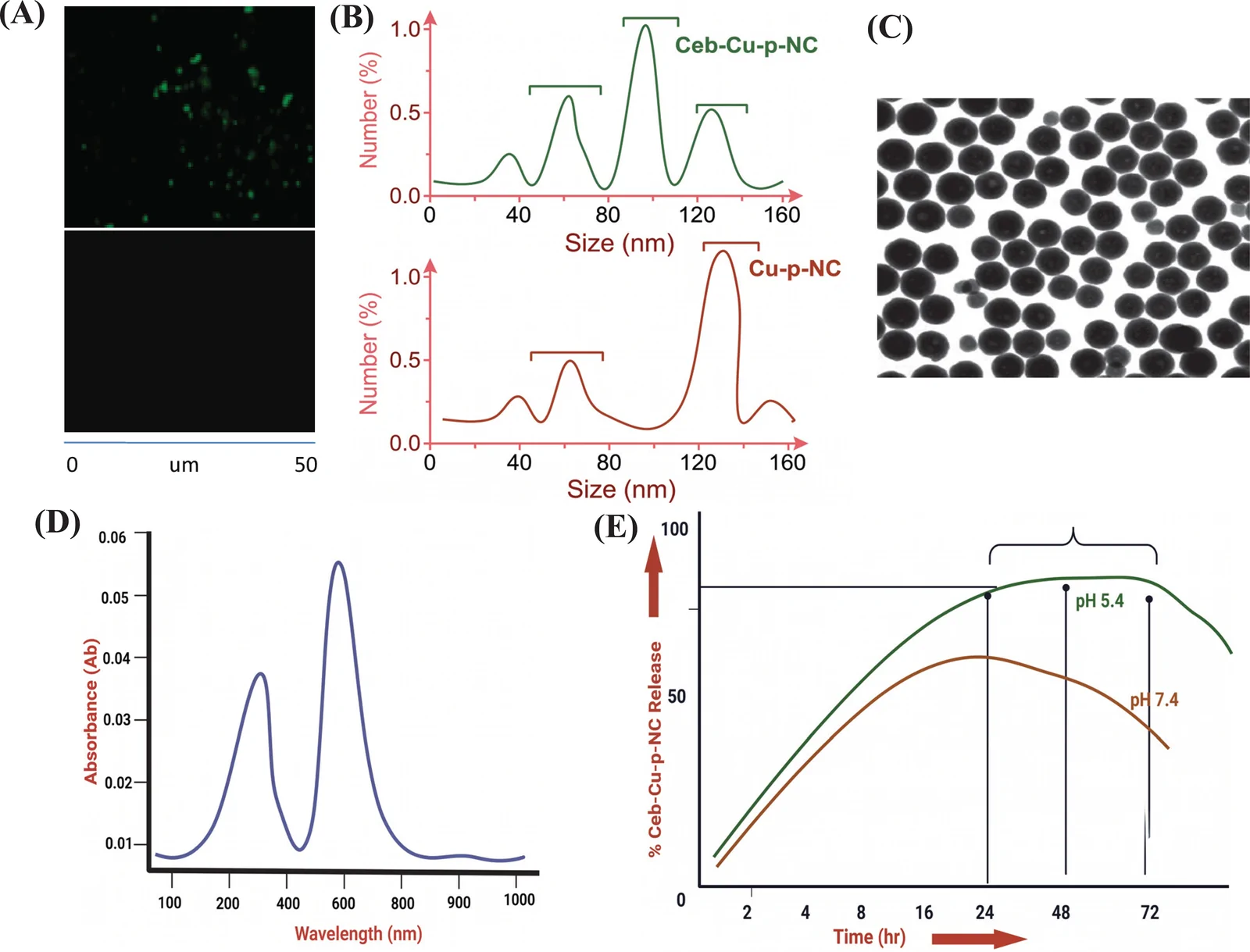
Characterizations of Ceb-Cu-p-NC **(A)** The green fluorescence was seen in the Ceb-Cu-p-NC, and no fluorescence could be detected in Cu-p-NC, **(B)** Particle size range, DLS, **(C)** TEM of Ceb-Cu-p-NC, with **(C.1)** map displays grain boundaries marked by UNet, with red-colored convex contours detected by CHAC. **(D)** UV wavelength and **(E)** In-vitro Ceb release from Cu-p-NC on both pH ranges.

Ceb-Cu-p-NC exhibits a notable entrapment efficiency of 82.5%, with in-depth examination revealing its stability and controlled release properties. In-vitro release studies were conducted under varying pH conditions (5.4 & 7.4) to replicate the extracellular physiological environment and the neutral pH prevalent in the tumor microenvironment. The release profiles of Ceb were meticulously analyzed at distinct time intervals up to three days (Fig. **2E**). Remarkably, Ceb-Cu-p-NC demonstrated stability under extracellular conditions, showcasing a substantial increase in Ceb release (76.80%) under near-neutral pH conditions. Drug release at pH 5.4 is significant as it reflects the acidic environment of tumors, enabling targeted delivery and reducing harm to healthy tissues [57]. The release behavior exhibited synchronicity, characterized by a slow initial release followed by controlled release from 9 to 72 hours, indicating a sustained and controlled drug release over a three-day period. This precise and orchestrated drug release profile positions Ceb-Cu-p-NC as a promising advancement in targeted cancer therapy, providing a sophisticated solution to challenges faced by current drug delivery systems for HNSCCs.

### 3.3 *In vitro* Cytotoxicity and Cell Viability Analysis

Assessing A-253 cell viability post-administration of free Ceb, Cu-p-NC, and Ceb-Cu-p-NC included staining with DAPI reagent, followed by fluorescence microscopy.

**Fig. (3):**
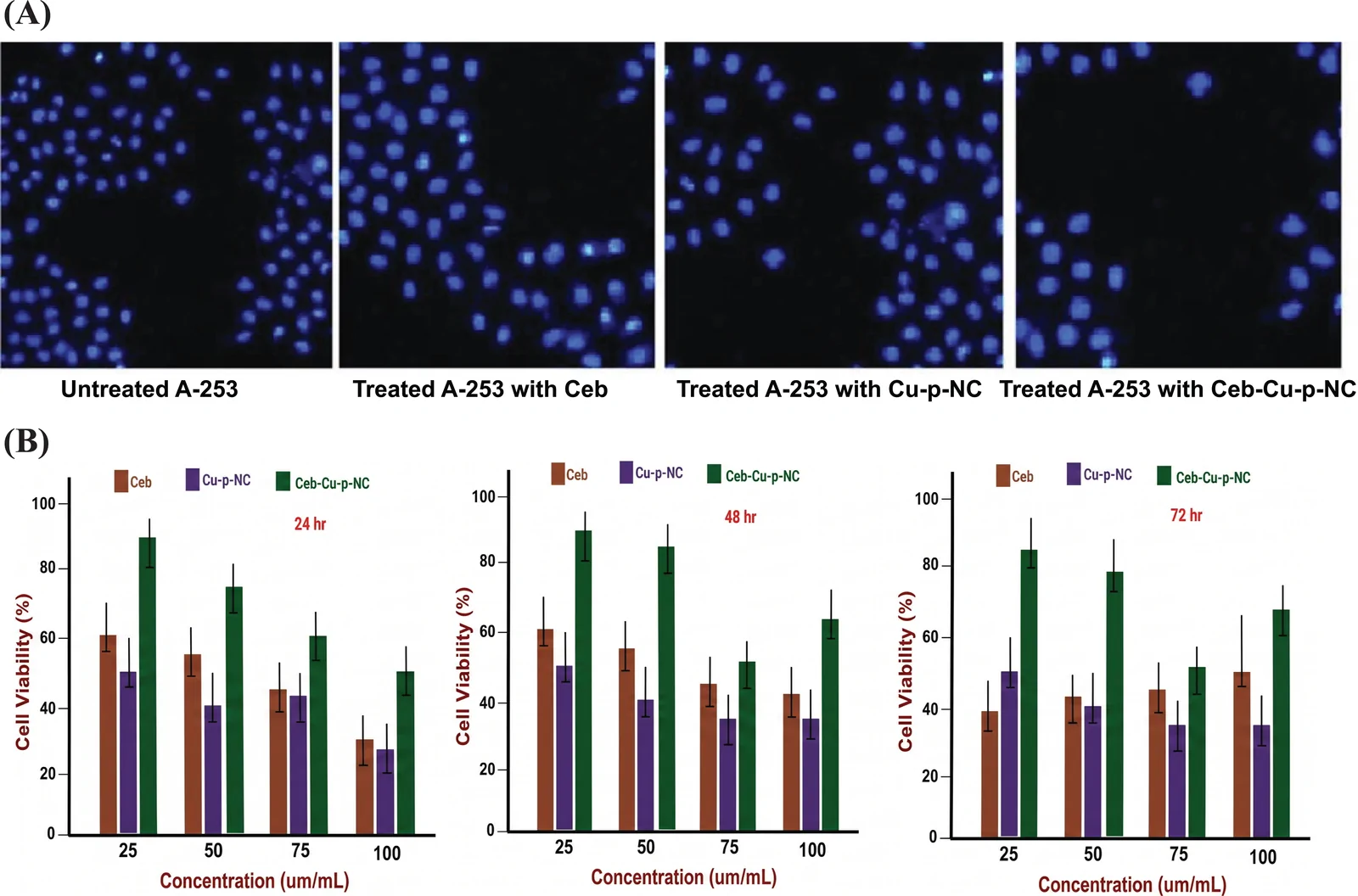
**(A)** Effect of varying concentrations of Ceb, Cu-p-NC, and Ceb-Cu-p-NC on A-253 cell viability at 24, 48, and 72 hours. Ceb-Cu-p-NC shows significantly higher anti-proliferative activity compared to Ceb (*p < 0.05). **(B)** Apoptosis and DNA damage in A-253 cells induced by Ceb, Cu-p-NC, and Ceb-Cu-p-NC, confirmed by DAPI staining after 48 hours of treatment with 100 µg/mL.

Fig. (**3A**) displays reduced fluorescence intensity in small hemispherical nuclei after treatment with free Ceb, Cu-p-NC, and Ceb-Cu-p-NC. Optimal doses of formulations resulted in a notable increase in apoptosis, with Ceb-Cu-p-NC demonstrating a more pronounced apoptotic effect in the A-253 cell line compared to free Ceb. In summary, 25ug/mL concentrations of Ceb-Cu-p-NC are capable of inducing a reduction in the viability of the A-253 cell line, indicating persistent DNA damage as a contributing factor. The IC50 values for Ceb incorporated within Ceb-Cu-p-NC were determined to be 27.55 mg/mL, 41.35 mg/mL, and 51.47 mg/mL after 24, 48, and 72 hours of exposure, respectively. Fig. (**3A** and **3B**) illustrate a discernible anticancer effect against the A-253 cell line for free Ceb, Cu-p-NC, and Ceb-Cu-p-NC, demonstrating a time and dose-dependent response. The distinctive cytotoxicity profile of Ceb-Cu-p-NC, attributed to its controlled and slow-release properties from 24 hr to 72 hr, surpasses that of free Ceb and Cu-p-NC, and shows the decreased cell viability of approximately 81.25% in A-253 cell exposure at different concentrations. This also indicates that 25 μg/mL is the optimum drug concentration of Ceb-Cu-p-NC for achieving up to 80% reduced cell viability (Fig. **3B**). Copper compounds, alone or in combination with other drugs, including those inhibiting topoisomerase, offer potent anticancer effects by influencing DNA regulation, cell cycle checkpoints, and apoptotic pathways, providing promising avenues for cancer treatment [58]. This underscores the efficacy of the Cu metal-based polymeric modification as an optimal formulation for targeted anticancer drug delivery, capitalizing on controlled release patterns that enhance therapeutic effects *via* specific pathways while maintaining higher intracellular concentrations [59, 60]. Folate-decorated nanostructured lipid carriers effectively co-deliver cisplatin and paclitaxel, showing superior anticancer efficacy in head and neck cancer in both *in vitro* and *in vivo* studies. These results underscore the potential of folate-decorated nanostructured carriers as an advanced dual-drug delivery system for enhanced therapeutic outcomes [61]. The controlled release of Ceb-Cu-p-NC results in higher cytotoxicity compared to free Ceb, making it a promising formulation for targeted anticancer drug delivery [62, 63].

### 3.4 Exploration of Cellular Apoptosis through Advanced Flow Cytometry Analysis

To understand the cellular responses, our investigation employed flow cytometry for a meticulous apoptosis assay, aiming to elucidate the substantial impact of various cell treatments [64]. The study examines the intricate mechanisms that govern how cells respond to targeted therapeutic treatments [65]. Remarkably, a notable surge in apoptosis became evident within the cohorts treated with free Cab and Ceb-Cu-p-NC, when juxtaposed against the untreated control group. Notably, the Ceb-Cu-p-NC-loaded group exhibited a significantly higher apoptotic rate compared to its free drug counterpart. The discerning analysis unveiled a noteworthy 32.5% apoptosis in the early stages and a more substantial 34.75% in the late stages within the Ceb-Cu-p-NC loaded group. Examining the late apoptosis chamber showed a notable 43.50% rate of apoptosis. These findings collectively underscore the heightened therapeutic efficacy and optimal anticancer effects attributed to Ceb-Cu-p-NC, as illustrated in Fig. (**4**). This investigation illuminates the potential of Ceb-Cu-p-NC as a promising candidate with a superior therapeutic index, poised to revolutionize anticancer treatments [66].

### 3.5 Investigating Cell Cycle Arrest

An essential aspect of comprehending the impact of Cab and Ceb-Cu-p-NC on cellular growth is the meticulous examination of cell cycle arrest and a detailed understanding of their molecular mechanisms [67]. This study investigated the dynamic changes in DNA content within A-253 cells over a 48-hour period, following treatment with Cab, Cu-p-NC, and Ceb-Cu-p-NC at a concentration of 100 μg/mL. The observed dosage-dependent response in A-253 cells revealed a noteworthy cell cycle arrest pattern, as depicted in Fig. (**5**). Notably, both Cab and the nanocarrier exhibited inhibitory effects on DNA synthesis, leading to a distinctive cell cycle arrest profile suggestive of traversal through mitotic division but subsequent arrest in the S phase [68].

Quantitative analysis revealed a notable increase in the percentage of cells in the S phase, from 14.54% to 27.67% for Cab and from 18.97% to 41.69% for Ceb-Cu-p-NC. This considerable increase signifies an elevated level of DNA damage, ultimately resulting in cell cycle arrest observed across G1, S, and G2/M phases. Corroborating this finding, a study reported calycosin inducing G0/G1 cell cycle arrest in treated cell lines *via* the AKT signaling pathway. Moreover, the observed S phase arrest is postulated to stem from the inhibition of cell cycle progression [69]. This inhibition is attributed to the nuanced anti-proliferative effects, finely modulated by the target-based release profiling characteristic of Ceb-Cu-p-NC. The intricate understanding of these cell cycle dynamics sheds light on the therapeutic potential and targeted efficacy of Cab and Ceb-Cu-p-NC in impeding cancer cell proliferation.

Future studies may focus on developing more effective drug delivery systems for head and neck cancer by utilizing advanced models and innovative treatment strategies. Patient-derived xenograft (PDX) models, which closely resemble human tumors, could help test drug effectiveness and resistance more accurately [70, 71]. Exploring genetic changes, such as mutations or deletions, will be important for understanding drug sensitivity. CRISPR/Cas9 technology could further aid in identifying new drug targets, thereby making treatments more precise [72]. A new type of cell death, known as "cuproptosis," has been discovered and is caused by excess copper in the cells [73]. This process involves 12 main genes and seven that support cell death, three that help prevent it, and two that control copper movement in the body. Learning more about these genes and how they function in cancer could help researchers develop more effective treatments for the disease in the future [74, 75]. Additionally, cuproptosis-based drug delivery, such as Cu-triggered nanotherapeutics using copper-based ionophores and nanoparticles [65, 66], shows a promise for improving cancer therapy [76, 77]. Drug delivery in cancer treatment faces challenges, including accurately reaching the tumor, preventing harm to healthy cells, and ensuring the drug remains stable and effective in the body [78]. Natural barriers, early drug breakdown, or quick removal from the body can reduce treatment success [79].

## CONCLUSION

In conclusion, this study highlights the promising potential of Ceb-Cu-p-NC as a nanoengineered drug delivery system for targeted treatment of HNSCC. The optimized formulation, characterized by a nanoscale size (85-120 nm) and favorable zeta potential, demonstrates efficient Ceb integration (34.92%) and high entrapment efficiency (82.5%). *In vitro* studies reveal controlled, pH-sensitive drug release, with significantly enhanced cytotoxicity (IC50: 27.55 µg/mL – 51.47 µg/mL) against A-253 cells compared to free Ceb and Cu-p-NC. Flow cytometry analysis confirms substantial apoptosis induction and S-phase cell cycle arrest, indicating potent anti-cancer activity. These findings underscore the therapeutic potential of Ceb-Cu-p-NC, offering a dual-targeted approach with improved drug stability and efficacy. With further preclinical and clinical validation, this innovative platform holds a promise for advancing HNSCC therapy, potentially improving patient outcomes through more precise and effective drug delivery. While these results are encouraging, the study is limited to *in vitro* analysis, and factors such as *in vivo* stability, biodistribution, and long-term safety remain to be explored. Further preclinical and clinical investigations are essential to validate its therapeutic potential and applicability in clinical settings.

## STUDY LIMITATIONS

This study is limited to *in vitro* evaluations using A-253 head and neck cancer cell lines, which may not fully replicate the complexity of *in vivo* tumor environments. Additionally, the sample size and scope of biological assays were restricted to preliminary analysis. Future studies should involve *in vivo* validation, pharmacokinetic profiling, and long-term toxicity assessments to better establish clinical translation potential.

## Figures and Tables

**Fig. (1):**
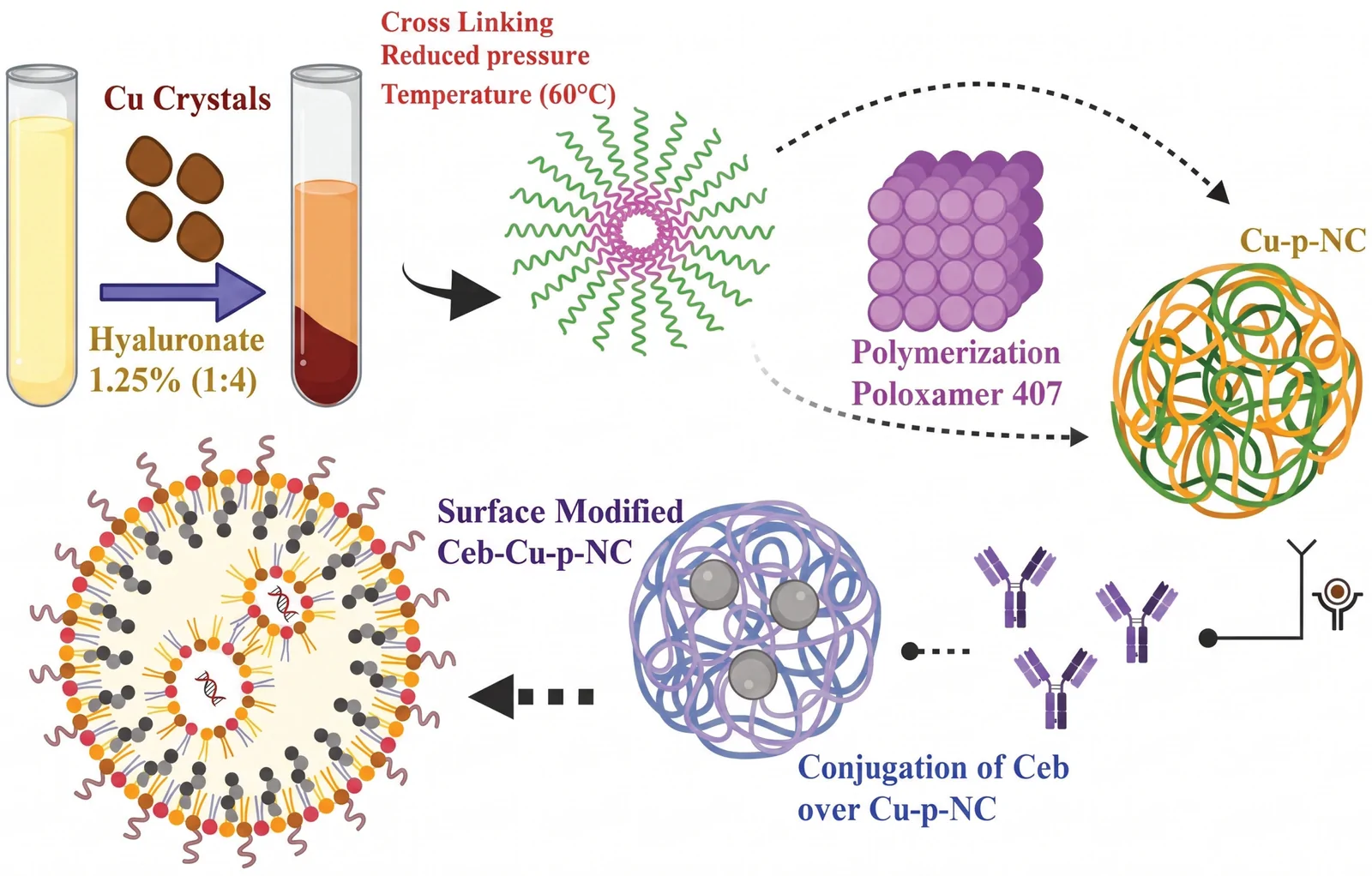
Surface-modified nanopolymerization of Ceb with Cu complex cross-linking.

**Fig. (4):**
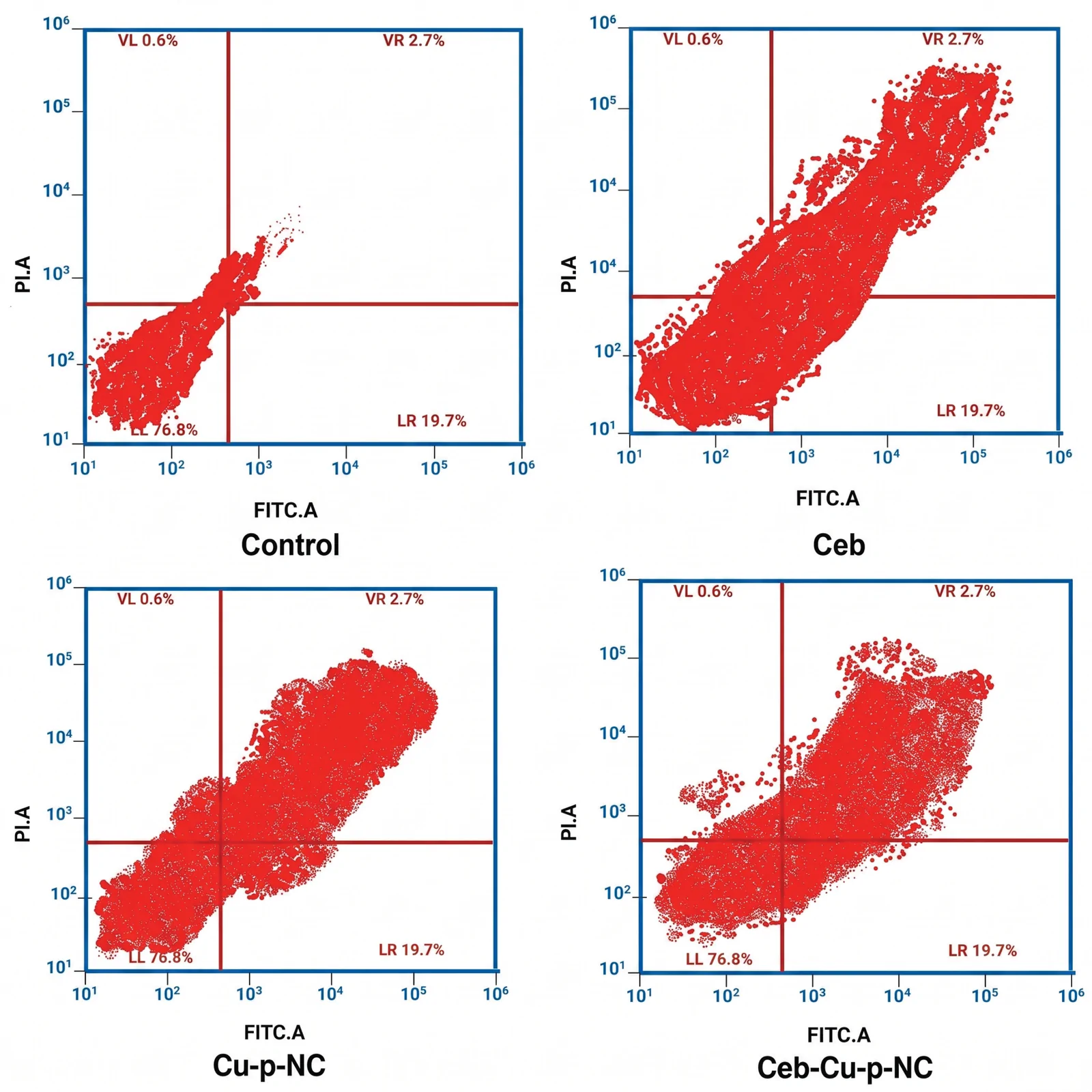
Flow cytometry analysis of A-253 cell responses to varying concentrations of Ceb-loaded Ceb-Cu-p-NC and free Ceb after 48 hours of treatment.

**Fig. (5):**
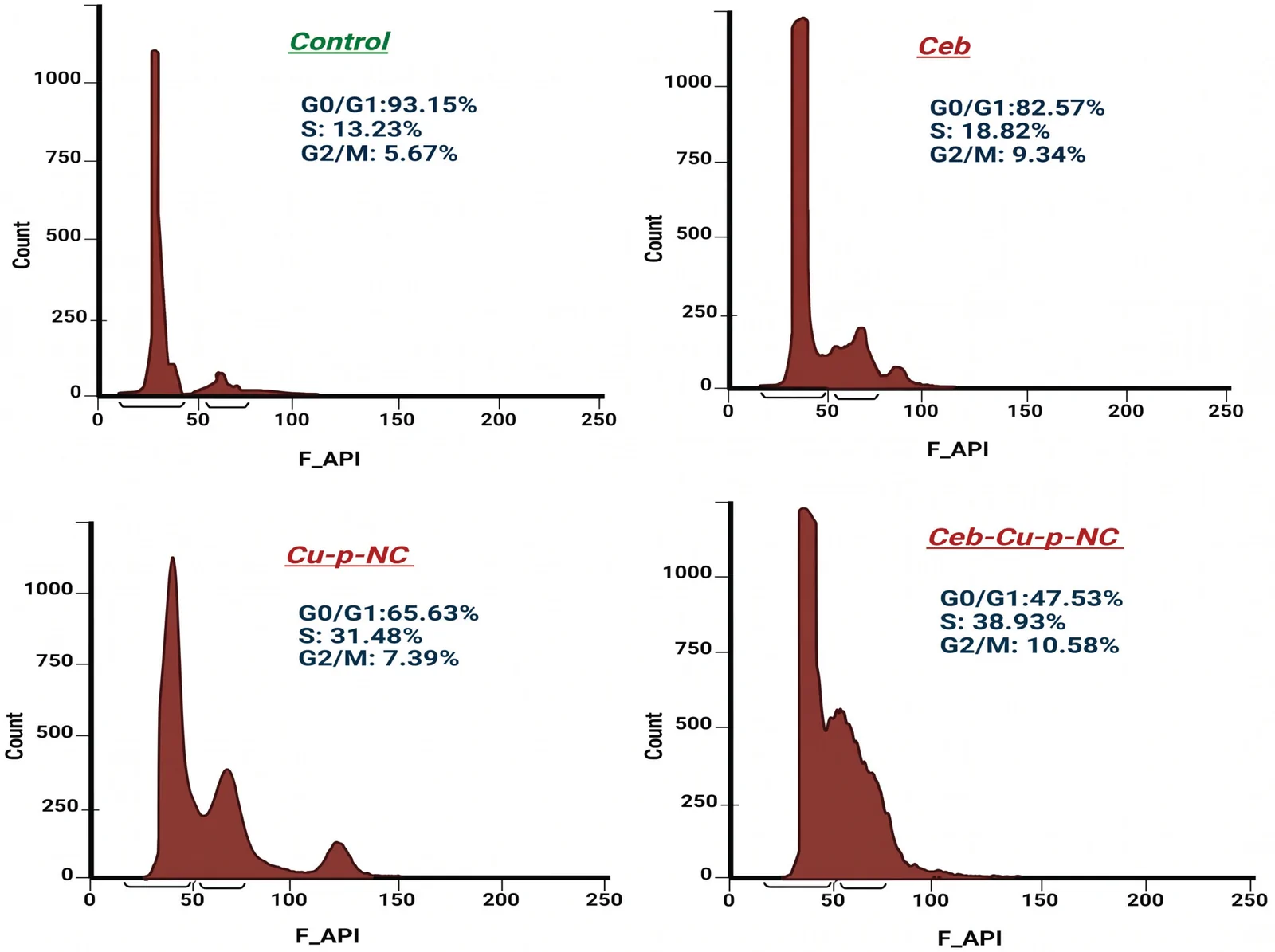
Flow cytometry analysis of A-253 cell cycle and DNA content after 48 hours of treatment with 100 µg/mL of Ceb-loaded Ceb-Cu-p-NC and free Ceb.

## Data Availability

All the data and supporting information are provided within the article.

## LIST OF ABBREVIATIONS

ANOVA: Analysis of Variance
ATCC: American Type Culture Collection
CAR-T: Chimeric Antigen Receptor T-cell
Ceb: Cetuximab
Ceb-Cu-p-NC: Cetuximab-conjugated Copper-polymeric Nanocomposite
Cu: Copper
Cu-p-NC: Copper-modified polymeric nanocrystal
CuCl_2_: Copper(II) chloride
DAPI: 4′,6-diamidino-2-phenylindole
DLS: Dynamic Light Scattering
DW: Deionized Water
EE: Entrapment Efficiency
EGFR: Epidermal Growth Factor Receptor
FBS: Fetal Bovine Serum
FDA: Food and Drug Administration
FITC: Fluorescein isothiocyanate
FTIR: Fourier-Transform Infrared Spectroscopy
GSH: Glutathione
HA: Hyaluronic Acid
HNSCC: Head and Neck Squamous Cell Carcinoma
HPV: Human Papillomavirus
IC_50_: Half Maximal Inhibitory Concentration
IgG: Immunoglobulin G
mAb: Monoclonal Antibody
MTT: 3-(4,5-dimethylthiazol-2-yl)-2,5-diphenyltetrazolium bromide
MWCO: Molecular Weight Cut-Off
Na_3_PO_4_: Sodium Phosphate
NaCl: Sodium Chloride
NC: Nanocarrier / Nanocomposite / Nanocrystal
NP(s): Nanoparticle(s)
O.D.: Optical Density
P407 / PLX-407: Poloxamer 407
PBS: Phosphate-Buffered Saline
PDI: Polydispersity Index
PG: Propylene Glycol
PI: Propidium Iodide
PS: Particle Size
qRT-PCR: Quantitative Real-Time Polymerase Chain Reaction
ROS: Reactive Oxygen Species
RPMI-1640: Roswell Park Memorial Institute Medium-1640
S-phase: Synthesis Phase (of Cell Cycle)
SD: Standard Deviation
SDS: Sodium Dodecyl Sulfate
SEM: Scanning Electron Microscopy
TEM: Transmission Electron Microscopy
UV: Ultraviolet
XRD: X-ray Diffraction
ZP: Zeta Potential
